# Mechanism of Allium Crops Bulb Enlargement in Response to Photoperiod: A Review

**DOI:** 10.3390/ijms21041325

**Published:** 2020-02-16

**Authors:** Muhammad Jawaad Atif, Mohammad Abass Ahanger, Bakht Amin, Muhammad Imran Ghani, Muhammad Ali, Zhihui Cheng

**Affiliations:** 1 Department of Vegetable Science, College of Horticulture, Northwest A&F University, Yangling 712100, China; jawaadatif@nwafu.edu.cn (M.J.A.); bakhtamin96@nwafu.edu.cn (B.A.); imran_pak@nwsuaf.edu.cn (M.I.G.); muhammadali@nwsuaf.edu.cn (M.A.); 2 Vegetable Crops Program, National Agricultural Research Centre, Islamabad 44000, Pakistan; 3 College of Life Sciences, Northwest A&F University, Yangling 712100, China; ahangerma@gmail.com; 4 College of Natural Resource and Environment, Northwest A&F University, Yangling 712100, China

**Keywords:** photoperiod, bulbing, phytohormones, FT gene, genotype, CO gene, allium

## Abstract

The photoperiod marks a varied set of behaviors in plants, including bulbing. Bulbing is controlled by inner signals, which can be stimulated or subdued by the ecological environment. It had been broadly stated that phytohormones control the plant development, and they are considered to play a significant part in the bulb formation. The past decade has witnessed significant progress in understanding and advancement about the photoperiodic initiation of bulbing in plants. A noticeable query is to what degree the mechanisms discovered in bulb crops are also shared by other species and what other qualities are also dependent on photoperiod. The FLOWERING LOCUS T (FT) protein has a role in flowering; however, the FT genes were afterward reported to play further functions in other biological developments (e.g., bulbing). This is predominantly applicable in photoperiodic regulation, where the FT genes seem to have experienced significant development at the practical level and play a novel part in the switch of bulb formation in Alliums. The neofunctionalization of FT homologs in the photoperiodic environments detects these proteins as a new class of primary signaling mechanisms that control the growth and organogenesis in these agronomic-related species. In the present review, we report the underlying mechanisms regulating the photoperiodic-mediated bulb enlargement in Allium species. Therefore, the present review aims to systematically review the published literature on the bulbing mechanism of Allium crops in response to photoperiod. We also provide evidence showing that the bulbing transitions are controlled by phytohormones signaling and FT-like paralogues that respond to independent environmental cues (photoperiod), and we also show that an autorelay mechanism involving FT modulates the expression of the bulbing-control gene. Although a large number of studies have been conducted, several limitations and research gaps have been identified that need to be addressed in future studies.

## 1. Introduction

The photoperiod is demarcated as the portion of light and dark hours in a diurnal cycle of 24 h [1]. The continual discrepancy in length of day, predominantly at better notches of latitude, is a dependable scale of the evolution of the spells and controls when the plant shifts its growing strategies and twitches forming bulbs. Arabidopsis is a facultative long-day plant, and flowers prior under long-day environments. Long days promote bulb enlargement. Nevertheless, day length does not distress the floral switch in tomato, which is a day-neutral plant. The photoperiod is believed to interrelate to stimulate bulbing [2,3]. Far-red light is also obligatory. In alliaceous crops, bulb initiation is exceedingly inclined by day length. Bulb swelling is attracted if the day length exceeds 12–15 h, which is liable on the cultivar [3]. Brewster [4] informed that garlic produces inadequate bulbs in warm, short-day lowland tropical areas, but in temperate zones, the bulb size is characteristically large. The photoperiod, similar to other ecological parameters, regulates the control of plants over inner indicators and alterations in hormonal profile. Endogenous gibberellins levels are enhanced with a long photoperiod, which has been shown to have a dynamic part in the bulbing of garlic and growing quantity of cloves [5]. There is variability in bulb characteristics, clove color, harvest, and aroma according to growing atmosphere and cultivar [6]. Present-day garlic cultivars are purified, and seed manufacture was testified only for a limited genotype in some sites. Garlic is frequently proliferated vegetatively, and adopting an early planting routine that is of adequate scope and eminence is decisive for accomplishing high produce yield [7]. The properties of photoperiod on progressive developments of bulbing in onion have been studied [8]. The onion cultivars of varied origin exhibited better bulbing under a long photoperiod (17 h·d^−1^). It has been reported that the degree of bulb growth over long photoperiods increases [9]. Under short-day environments, onion plants formed new plants indeterminately deprived of bulb development, whereas at longer day lengths, bulbs were formed [10]. Photoperiods under 11 h muted bulbing in the two tropical onion varieties, and bulbing was enlarged gradually with as the day length surged [11].

The FLOWERING LOCUS T (FT) gene family in Arabidopsis encrypts the transportable flowering indicator formed in the leaves that is responsible for generating bulb development in the subversive stolon, thereby providing a vital innovation about the way bulbing is controlled. Further research in onion exhibited that diverse FT genes similarly control bulb enlargement, stressing the evolutionary-focused maintenance of FT proteins as key producers of the bulb alteration. This too influences the offshoot meristems at the axillary buds and vascular cambium etches for this progressive changeover. FT-like genes switch onion bulb development; however, unlike Arabidopsis FT, it is not a long-distance indicator. These fallouts and the preceding information indicate that FT-like genes activate bulb development in *Allium*, proposing the turnover of all controllers of bulb advance in varied taxonomy [12]. Furthermore, expression of the LONELY GUY (LOG1) gene, encrypting a cytokinin (CK)-activating enzyme, converses axillary tomato meristems to an aptitude of de novo creation of tuber-like organs [13], recommending that CKs may have a purpose as extensive controllers of storage-organ formation in plants. This research work displays that the ectopic expression of TLOG1, which is a cytokinin-activating gene, gives axillary tomato meristems the capability to produce midair tiny tubers that reflect the involvement of cytokinin in initiating the storage organ development in a non-tuberizing plant. This makes an outline for researching tuber beginning grounded on the unexpected similarities in the parameters of tuberization among axillary meristems and tempted stolons.

Plants respond to different seasons to initiate evolving strategies at specific times of year. The photoperiod controls several evolving developments in plants. Genomic studies have revealed the fundamental responses to alterations in photoperiod and the generation of a vigorous cyclic response. Current advances in plant genome analysis have established the variation in these controlling structures in several crops. Plants are visible to a sturdy rhythm of day and night due to the rotation of the earth around its own axis. This rhythm varies throughout the year, and the more distant an area is from the Equator, the sturdier these variations are, therefore demanding the adaptation of life procedures to this altering rhythm. The adjustment sequence and thus the synchronization of frequent biological and progressive developments with the ecological sequences is understood by an inner circadian clock. Throughout evolution, a quantity of plant classes has achieved the skill of distinguishing their leaves, stems, or roots into storage organs (bulb), as observed in garlic and onion. The formation of these bulbs is attracted throughout drought and freezing conditions that pacify plant feasibility, acting as a mechanism for asexual propagation that delivers a survival strategy to the plant. As such, these bulbs are continuously dormant in soil throughout divergent cold and dry times, after which they are sown in the following term and produce a healthy plant. Early growing of the new shoot is liable on the metabolic properties that have accumulated in these bulbs, generally in the formula of starch or soluble sugars, which makes them an outstanding caloric supplementation to the human nutritional requirements. An iterative breeding collection for bigger bulbs and form to diverse latitudes could improve the recent cultured genotypes, which is of high financial significance and tactical in equilibria of food safety. Therefore, understanding the mechanisms recycled by the plant to signal the distinction of these bulbs is a vital area to see the nutritious stresses of the increasing world population, and it is also an ultimate query in developing environmental skills.

## 2. Photoperiodic Control in Bulb Enlargement

A bulb is a vegetative growing point or an unexpected flowering shoot. The base is from a reduced stem, and plant growth occurs from this basal plate. Root development forms from the underside of the base and new stems and leaves form from the upper side. A long photoperiod is crucial for bulb initiation and growth in *Allium sativum* [2,3,13,14,15,16,17]. Although the precise structures are uncertain, the photoperiod and the mechanism for bulbing have been crucial limits for the progressive development of bulbs or the strategy of agricultural systems. A detailed understanding of the properties of ecological environments (photoperiod) on bulb growth would increase our information of the bulb developments and enable the production of a continual stock of bulbs. The photoperiod expressively impacts the propagative developments [7]. The bulbing and cloving are inclined by the day length to which the dormant cloves or budding plants are subjected before bulbing starts [18]. At large, low early temperatures followed by long days are crucial for the development of bulbs and cloves. At the same time, the struggle for resources by the parallel developing bulb regulates the providence of bulbing [19]. In onion bulbs, a healthy sink in the initial bulb growth phases pacifies the evolution and divergence of the new inflorescence with subsequent drying of the flower stem. Henceforth, it was planned that the stimulation of the photoperiod on bulb growth should be meticulous in the background of the corresponding but uncertain growth of bulbs in garlic [7] (Table 1).

Up to the present time, the result of photoperiod on bulb development has been researched for some Alliums. The bulb enlargement of onion and its subsequent development were influenced by the photoperiod, and bulbing was encouraged by long days. Furthermore, in some cultivars, bulbing only occurred once double thresholds of a least thermal period of 600-degree days and a photoperiod of 13.75 h were achieved [20,21,22,23]. However, to the best of our information, the risky conditions for bulbing and the alteration in the indicator ingredient through this development inside *Allium sativum* have attracted limited investigation reflection, apart from the research of Kamenetsky and Rabinowitch [24], Mathew et al. [7], Wu et al. [16], and Atif et al. [2]. The properties of irradiance on crop growing and interrelations among growth and the developing procedures of bulbing in onions have been studied [8]. Findings with respect to light spectral eminence have revealed that the photoperiodic switch of bulbing is a high-irradiance response of the phytochrome scheme [25]. Far-red light, which turns over phytochrome A, encourages bulbing most efficiently throughout the central portion of an 18-h inductive photoperiod, necessitating around five spells as abundant energy for the similar response at the start and finish of this photoperiod [26]. Furthermore, the lesser the red to far-red proportion, the better the raise of bulbing in a certain photoperiod [27,28,29]. Red light unassisted or useful proximately after separate far-red radiation repressed bulb enlargement in onion [30]. A lower red to far-red ratio rushes bulb measure at the beginning and maturity as the leaf area index rises. The light intensity, light quality, and additional features interrelate with day length to affect the bulbing response of onion cultivars. In warm weather and bright days, onions bulb at shorter day lengths than the cool and cloudy days [31,32]. Environmental factors, such as for example the day length, affect bulb enlargement and taste eminence in onion [33]. In onion, light range and quality impacts bulb formation and quality [34,35,36,37,38,39,40,41]. The bulb initiation and growth of further *Allium* species including garlic have been shown to be influenced by day length, temperature, and carbohydrates [42,43]. Earlier investigators testified that plants treated with shorter day lengths than they needed will form solitary leaves with poor bulbs [44], and in some conditions, thick bulb necks might too occur [45]. Equally, untimely bulb development, bulb growth, and maturity tolls rise after plants are subjected to longer day lengths than they require, which results in small bulbs and small yield [11,46].

## 3. Phytohormonal Control of Bulb Enlargement

**Figure 1:**
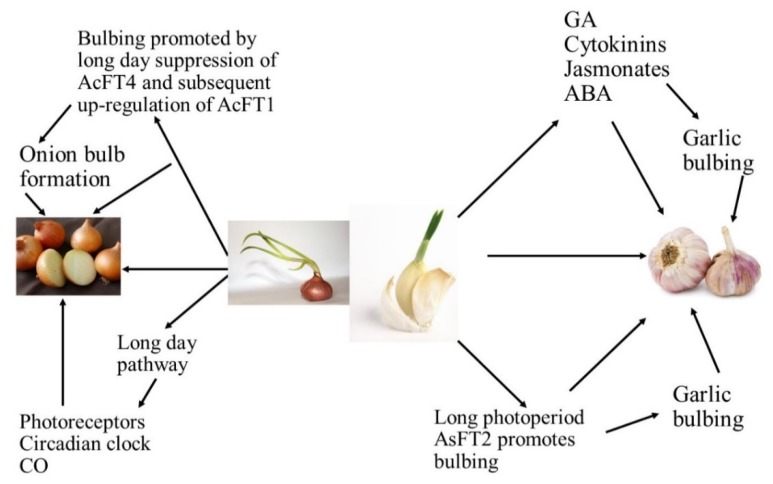
Schematic representation of role of photoperiod, FT-like genes, and phytohormones in *Allium sativum* and *Allium cepa* bulb formation.

Phytohormones are considered the most important endogenous substance for modulating physiological and molecular responses, and they are a critical requirement for plant survival as sessile organisms. Phytohormones act either at their site of synthesis or elsewhere in plants following their transport. The examination of the paraphernalia of photoperiod on bulb growth will offer vision into the mechanism of bulbing and environmental tools (adaptable photoperiods) for developing parameters. The directive of bulbing is comprised of intricate evolutions determined by an intricate system of signaling pathways. In order to improve propagation ability and bulb production through horticultural practices, regulating environmental conditions (photoperiod) and phytohormones are the most effective ways, which have a key role in the development of bulbs [7,15,47,48]. Hormonal balance has a large effect on storage organ formation and development. The role of hormones in the sprouting of garlic cloves has been demonstrated. In addition to environmental cues, such as photoperiod, bulbing is also controlled by endogenous signals, including phytohormones level and plant age [49,50] (Table 1) (Figure 1).

### 3.1. Gibberellic Acid

Endogenous gibberellins were found at high levels in the storage leaf during clove sprouting, and they also inhibited dormancy induction in whole tulip bulbs [51,52,53]. Long photoperiods are identified to improve the levels of endogenous gibberellins, with substantial flower sprout divergence [5,7]. Various experiments have revealed that gibberellic acid (GA) might partially or completely substitute vernalization [54,55,56]. Surge is detected in the endogenous GA levels of long-day or biennial plants throughout the development of floral initiation. However, GA is a well-organized inhibitor of bulb growth [56]. The application of exogenous GA on garlic revealed that exogenous GA subdued the rise of the bulb produce. It was likely that GA did not turn reliably on the inhibition of bulbing, in its place attracting the outbreak of a “bulbing reserve substance” [57,58]. GA_3_ application could increase shoot weight in carrot [59], seed yield in lettuce [60], and fruit weight in pear [61] and plum [62]. Earlier reports have revealed that the application of GA_3_ increased bulb weight in onion under deficit irrigation [63]. On the contrary, a few results have shown that the application of GA_s_ caused a decline in tuber production in potato [64]; fruit production in sweet pepper [65], grape [66], and pear [67]; and root weight in carrot [59]. In potatoes, it was testified that GA_3_ treatment improved the number of tubers per plant [68]. The application of GA_3_ also encouraged tillering in welsh onions [69], encouraged airborne tubers development in turnip [70], and promoted shoot branching in *Jatropha curcas* [71]. GA was also used to progress fruit morphological characteristics (skin color and firmness) and nutritive quality in apples, bananas, plums, and sweet peppers [72,73,74] (Table 1).

Gibberellins are imperative phytohormones that control many evolving developments in plants [75]. According to Liu et al. [75], GA_3_ treatment intensely encouraged lateral bud development but repressed the growth of plants and bulbs, and lateral bud formation doubled in garlic plants treated with GA_3_. Bulb nutritious qualities were enhanced by applying GA_3_. Briefly, the number of cloves and whorls per bulb improved with a higher concentration of GA_3_. After application of a low concentration of GA_3_, the number of cloves per bulb and the soluble sugar content were expressively improved, but the mean bulb weight was significantly reduced for plants treated with a high concentration of GA_3_ [75]. Gibberellins (GA_s_) are a growth-encouraging phytohormone [76,77,78], and the application of GA_s_ might significantly increase plant weight in cauliflower and sweet peppers [65,79,80]. In addition, it has been stated that a high concentration of GA_3_ and high frequency of application GA_3_ inhibit lettuce growth [60] and decrease fruit weight in plum [81]. The application of GA_3_ decreased root, tuber, fruit weight, and yield in other crops as well [59,64,67]. Previous reports have revealed that the application of GA_3_ had a positive and negative effect on the organ nutritive eminence of potatoes, sweet peppers, and sweet cherries [65,82,83]. GA_3_ can significantly induce lateral bud formation and increase the clove number per bulb, which improves the reproduction efficiency in garlic. However, the physiological and metabolic changes of garlic plants under GA_3_ treatment are unknown [75]. Lately, many genes for lateral bud development have been specified in other plants, and future work will focus on changes in gene expression and protein interaction in garlic plants after exogenous GA_3_ treatment [84,85,86,87]. Liu et al. [88] also studied the effect mode and time of application of GA_3_ on garlic plant architecture and bulb structure. Here, they investigated the effect of both soaking seed cloves in GA_3_ solution and injecting plants with GA_3_ on plant growth and bulb development in garlic. They detected that soaking seed cloves in GA_3_ solution induced secondary plant (equated with tiller or lateral branch) development and expressively increased the incidence rate of secondary plants, clove numbers per bulb, and bulb weight. Clove number per bulb and bulb weight were sharply increased by the application of GA_3_. Exogenous GA_3_ induced the axillary bud formation of garlic via the changes of soluble sugar content and soluble protein content in the stem [88]. Exogenous gibberellic acid (GA_3_) induced axillary bud formation and promoted the growth of lateral branches in tomato [89], potato [90], cherry tree [91], *Jatropha curcas* [71], and welsh onion [69]. The application of exogenous GA_3_ not only intensely increased clove number per bulb but also changed bulb morphology [75]. Currently, the application of exogenous GA_3_ is considered a new means for improving propagation competence and garlic yield. These results highlight the status of exogenous GA_3_ as a start of axillary bud development [88]. According to Liu et al. [88], soaking seed cloves with GA_3_ solution and injecting plants with GA_3_ caused a substantial increase in the incidence rate of secondary plants, clove numbers per bulb, and bulb weight, which can be used for improving propagation efficiency in horticultural practice.

### 3.2. Abscisic Acid

Abscisic acid (ABA) normally plays a vital part in plant resistance to biotic or abiotic stresses. It was expected that ABA plays an important role in the initial phase of plant bolting [92,93]. Su [94] described that endogenous ABA influences the flower shoot distinction of welsh onion (*Allium fistulosum* L.), which improved expressively and reduced subsequently after the flower bud variation. According to the outcomes of Wu et al. [16], ABA also exhibited a surge under the longer photoperiod. The increased endogenous ABA might accelerate the maturation process of garlic plants, which leads to the shorter growth period under longer photoperiods. However, when investigating the result of each factor and their interactions, ABA responded otherwise to the photoperiod; the ABA level was increased by a shorter photoperiod [16]. Abscisic acid (ABA) was recommended to play a key part in the whole growing development of garlic [51]. In Dutch iris, under bulb-inducing environments, endogenous ABA levels increased [95]. ABA were related to temperature regulation in dormancy initiation [96]. ABA was also found to play a crucial part in improving plant lenience to cold, as well as inducing leaf senescence in wheat and barley [97]. A further development that is facilitated by phytohormones was found in apical dominance control by balanced hormonal signaling between auxin, cytokinin, and the recently discovered strigolactones in grasses [98]. The role of ABA in the deep dormancy of seeds, tubers, and bulbs has been recommended in several investigations and reviews [99,100,101,102]. A variation in ABA level was also confirmed in onion (*Allium cepa* L.), where the level of ABA was higher during the dormancy period and decreased when dormancy broke. Furthermore, later research established that exogenous ABA delays sprouting in *Allium wakegi* plants [103]. ABA’s role in the later growth stages, the sprout regulation of potato tubers, and the effect of sucrose on its level have been proposed [102]. However, cold stress has been shown to cause the accretion of ABA in plants as part of their defense mechanism [104]. According to Rohkin Shalom [48], significantly higher ABA levels were observed in garlic cloves stored at warm versus cold temperatures, signifying that warm temperatures are a more effective sprouting inhibitor in garlic cloves (Table 1).

### 3.3. Indole Acetic Acid

Indole acetic acid (IAA) exhibits inconsistent results, decreasing with rising flower bud variation degree and then growing expressively throughout the bolting procedure of welsh onion [94]. It is normal to accept that IAA derivatives have been affected by environmental conditions [105]. Although IAA and ethylene enhanced bulb development, rare research reports are available discussing role of abscisic acid (ABA) on bulbing [16,106]. Auxins have long been occupied to play a regulatory role in potato (*Solanum tuberosum* L.) tuber growth. Endogenous auxin levels were found to be high just prior to and during stolon swelling, after which auxin levels steadily decreased [107]. Moreover, Xu et al. [56] detected earlier tuberization when IAA was applied to single node cuttings in a tuber-inducing medium. Instead, tuber formation was totally inhibited by high concentrations of IAA. In potato, only a single Aux/IAA (StIAA) [107] and ARF protein (ARF6) [107] have been designated up to the present time. StIAA expression levels increased after fungal infection, wounding, or the application of auxin [107]. Arf6 expression levels are reduced in the apical meristem of the stolon tip at the tuber beginning and development, and they are induced during meristem instigation in dormant tuber buds [107] (Table 1).

### 3.4. Zeatin Riboside

Zeatin riboside (ZR) was reported to have an enhancing effect on plant bolting [94]. According to Wu et al. [16], ZR levels increased as the photoperiods increased. Cytokinin (CTK) was a bulbing originator but had no noticeable impact on bulb widening [16]. Exogenous cytokinin was revealed to induce early differentiation and cell division in developing leaves [51]. Wybouw and De Rybel [108] highlighted how cytokinin influences growth and development in plants. In addition to being the main determinants of shoot development, cytokinins have also been implicated in many aspects of root development. For example, this is very clear when looking at the wide range of root-related phenotypes of biosynthesis, perception, and signaling mutants [109]. The Arabidopsis root shows clear bilateral symmetry within the vascular tissues, with a central xylem axis flanked by two phloem poles and intervening procambium cells. In this diarch setup, there is high auxin signaling in the xylem cells, whereas neighboring procambium and phloem cells display high cytokinin signaling. This bilateral character of the vasculature is a consequence of a tight interplay between auxins and cytokinins because auxin signaling in xylem cells induces AHP6, which in turn represses cytokinin signaling [110]. In addition, cytokinin signaling in procambial cells affects auxin efflux through PIN-FORMED (PIN) protein expression and localization [111]. Mathematical modeling suggests that this interplay is sufficient to achieve bilateral symmetry within the vasculature [112,113,114,115]. Cytokinins are tightly linked to vascular development because classical mutants in the signaling pathway such as wooden leg (ahk4/cre1/wol) and ahp6 were identified because of their vascular defects. In the past few years, the heterodimeric transcription factor complex formed by the bHLH transcription factors TARGET OF MONOPTEROS 5 (TMO5) and LONESOME HIGHWAY (LHW) has emerged as an important regulator of vascular proliferation [116,117,118,119]. Cytokinins also regulate root development in a longitudinal sense. In the root, cytokinins control root meristem size by limiting auxin activity in the transition zone. Here, cytokinins repress auxin activity through the direct induction of SHY2/IAA3 by ARR1 and ARR12. SHY2/IAA3 acts as a repressor of ARF activity and negatively regulates PIN proteins, leading to auxin redistribution [120,121]. Plant–microbe interactions between plants from the Fabaceae family and nitrogen-fixating bacteria lead to the formation of specialized plant structures called root nodules, and this process is strongly linked to cytokinin signaling. In *Medicago truncatula* and *Lotus japonica*, the inoculation of nitrogen fixating bacteria leads to an increase in cytokinin biosynthesis and signaling within the affected roots [122,123,124,125] (Table 1).

### 3.5. Jasmonic Acid

Jasmonic acid (JA) and connected amalgams are extensively dispersed amongst higher plants and play significant parts in the directive of plant growth [126,127]. Jasmonates have been shown to be effective inducers of nonsexual storage protein gene expression [128] and proteinase inhibitors of resistance proteins [129,130]. It is usually supposed that the bulbing course is controlled by the equilibrium amid the ‘bulbing hormones’ and GA [131,132]. Regvar et al. [133] and Žel et al. [134] testified that JA improved the bulb growth in vitro in absorptions from 1 to 10 μM and recommended that JA could play a significant role in the development of storage tissues in plants, for instance bulbs. Nojiri et al. [135] observed that bulbing was complex in the disorder of microtubules and suggested that jasmonic acid (JA) and methyl jasmonate (MeJA) were candidate bulbing hormones due to their microtubule-disrupting activities and extensive transport in higher plants (Table 1).

### 3.6. Salicylic Acid

Salicylic acid (SA) also has a significant role in garlic bulb development, and MeJA probably boost the endogenous SA content of garlic plant, therefore refining bulbing. Cytokinins have also been shown to have a part in tuberization by persuading local cell propagation throughout the initial tuberization start. Tuber development can be tempted in stem node carvings cultured in media with a high absorption of sucrose in the occurrence of these hormones. There was a decrease in the number of tubers per plant in transgenic lines ectopically articulating the cytokinin oxidase (CKX) inactivating enzyme [136]. It is significant to answer what is incomparable to the potato plant and can reverse potatoes with the ability to technique underground tubers as a vegetative propagation mechanism. Curiously, ectopic expression of the tomato LONELY GUY 1 (TLOG1), a cytokinin biosynthesis gene that alters in-active sugar-coupled cytokinin into their free active formula, has been revealed to consult juvenile tomato axillary meristems with the skill to form tuber-like structures [13] (Table 1).

## 4. Genetic Regulation of Photoperiod

Cultivars grown at diverse latitudes required a least day length for bulbing, and cultivars are classified on this into short-day (SD), intermediate, and long-day (LD) categories. The short-day cultivars procedure bulbs at low latitudes wherever the day length is close to 12 h, whereas intermediate ones grow bulbs at mid-latitudes wherever the day length lies between 12 and 16 h, and long-day cultivars initiate bulbing at high latitudes wherever the day length close or above 16 h [137]. Bulbing erstwhile to bulb anticipation is a thoughtful flaw instigating leading yield damage [138]. In the 21st century, notable development has been done in cereals and several additional crops to recognize the homologs of the Arabidopsis diurnal clock and additional flowering linked genes. All these investigations reflect that flowering genes are preserved in monocots and dicots to control flowering paths [139,140]. There is a sum of counterparts amongst the photoperiodic switch of onion bulb enlargement and flowering [12,141]. As with flowering, day length insight perhaps happens in the leaves, while Brewster [4] observed that the retort is in the meristem, signifying that a moveable indicator with characteristics similar to FT might be involved. For instance, as with the long-day initiation of flowering, bulbing requires the availability of light with a considerable unit of far red in the second half of the long day, concluding the dimension of the length of daylight in the evening via the chance of a daily controlled protein and its stabilization in the light involving phytochrome [4]. The financial, social, and nutritional implications of onion are a problematic topic for genetic investigation due to its incomplete genomics properties and an absence of agreed reference resources [142,143] (Table 1).

A difference among garlic cultivars in bulbing and responses to environmental indicators is predictable and is furthermost probably alike to what mutual happens in other Allium crops [4,144,145,146]. In alliaceous crops, bolting is dependent on ecological signs, i.e., long photoperiods (lily) or high (tulip, narcissus) or low (onion) temperatures [4,144,145,146]. Long photoperiods are vital for floral scape elongation. Generally, bolting-type garlic plants need 30–40 days under 0–4 °C or 50–60 days under 10 °C at the four-leaf age for vernalization. Later in that phase, a long photoperiod (≥13 h) and higher temperature (25 °C) is mandatory for the bulbing of garlic [14,15,147]. Day length consideration places a remarkable barrier on breeding agendas as the choice typescripts that initiate in onions from a specific day-length cluster cannot be moved to alternative day length collection by cross breeding, since the precise day-length response of the offspring is unidentified. Furthermore, crossing onions with different day length necessities is problematic, as the progeny will be compromised. Classifying the genes accountable for the day-length obligation of bulb enlargement will help understand the foundation of the alteration, which is significant for familiarizing new cultivars for evolution and progress at diverse latitudes. Whole genomes have been sequenced for numerous species, for example *Arabidopsis thaliana* [148] and the crops rice (*Oryza sativa*), maize (*Zea mays*), wheat (*Triticum aestivum*), and legumes such as soybean (*Glycine max*) and barril medic (*Medicago truncatula*) [149,150,151,152,153,154]. However, because of the limitations regarding evolving, maintaining, and switching genetic brands, there are low records of genetic researches for *Allium* development [155]. *Allium* bulb enlargement is reliant on day length, and it is therefore similar to the day-length retort of flowering [141]. In contrast to the information added regarding the photoperiodic regulation of flowering, relatively little is recognized about the genetic parameters of bulb development [12,141].

## 5. Gene Expression and Bulb Enlargement

**Figure 2:**
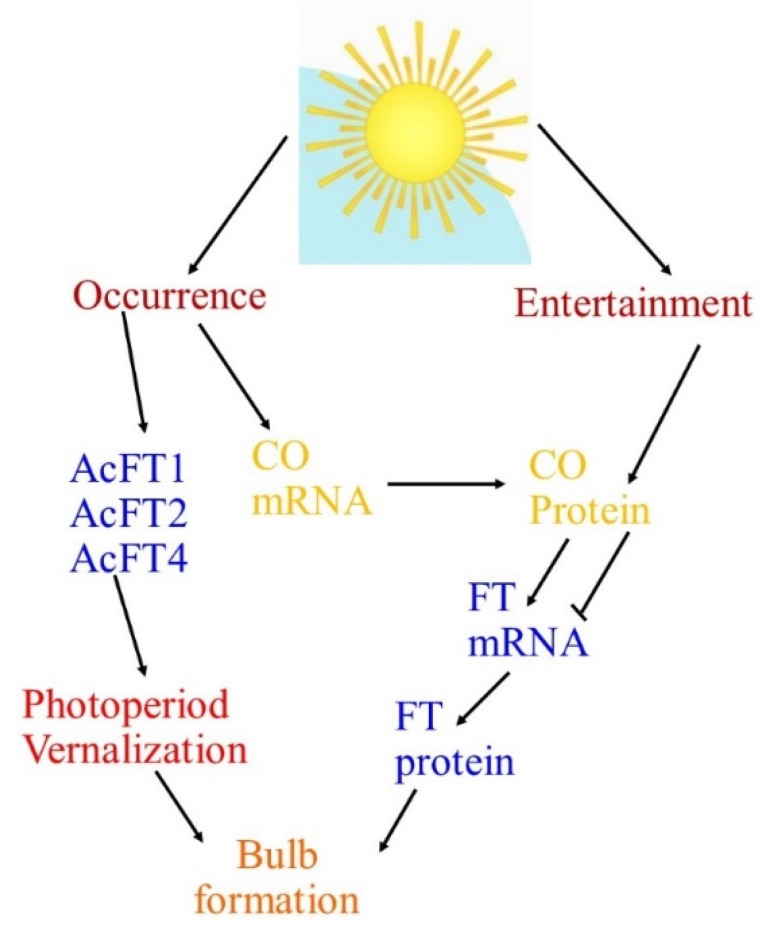
Schematic representation of photoperiodic responses. Model for regulation of bulbing transitions. Light entrains the circadian clock regulating CO mRNA expression. Light entertainments over an external occurrence mechanism to alleviate CO protein, which encourages or obstructs the expression FT mRNA. FT protein, form in the leaves, journeys through the phloem to distant locations of accomplishment with the apical or basal meristems or underground stolons (bulbs).

Gene expression is the process by which information from a gene is used in the synthesis of a functional gene product. These products are often proteins, but in non-protein coding genes such as transfer RNA (tRNA) or small nuclear RNA (snRNA) genes, the product is a functional RNA [156,157,158,159,160,161,162]. In onion, the physiology of the bulb beginning was precisely defined by Mettananda and Fordham [163]; photoperiodic conditions mark bulb beginning, similar to the photoperiodic switch of flowering in other species [20,164]. A topical investigation discovered that the flowering genes of Arabidopsis that have elaborate day-length responses are functionally preserved with respect to those intricate in onion bulbing [141]. An investigation of several species has discovered that FT-like proteins act throughout developing courses, such as for example the termination of meristem development and tomato produce [165,166], tuberization in potato [167], termination development in poplar trees [168], plant architecture in maize [169], stomatal control [170], and multiplicative architecture in Arabidopsis [171]. Hereafter, it was meticulously possible that the genes leading photoperiodic flowering also control bulbing [12]. Although biological trials have discovered the standing of plant age, light quality, and photoperiod for the initiation of bulb formation [20,163], the “hormones” directing this process are not yet known. It has been lately stated that the FT protein, whose expression is promoted by vernalization over repressor release, panels not only flowering but also bulb formation in onion [172] and tuber formation in the Solanaceae [173]. Several key genes are involved in circadian regulation, where the clock derives the rhythmic expression of key genes such as FLAVIN-BINDING, KELCH REPEAT, F-BOX (FKF1), GIGANTEA (GI), and CONSTANS (CO). FKF1 and GI promote CO expression [174], and this CO positively regulates FLOWERING LOCUS T (FT) [175]. Then, the FT protein is translocated to the apical meristem through the phloem and forms a FT/FD (FLOWERING LOCUS D) complex [176,177,178,179,180]. This compound triggers the APETALA 1 (AP1) and suppressor of overexpression of CONSTANS 1 (SOC1) genes, which triggers LEAFY (LFY) gene expression and causes flowering at the floral apical meristem in Arabidopsis [181,182,183]. The expression of GI, FKF1, and ZTL homologs under short-day and long-day environments was observed using quantitative reverse transcription-PCR (qRT-PCR), where the results presented that key genes—namely GI, CO, and FT—controlling photoperiodic flowering in Arabidopsis are conserved in onion, and a role for these genes in the photoperiodic control of bulb initiation is projected [163] (Figure 1 and Figure 2).

Bulbing is an alterable development, and plants grown under inductive environments promote bulb formation, but if they are moved to non-inductive condition, they quickly return to vegetative growth [8,184]. Bulb instigation can be distinct as the theme at which the “bulbing ratio”—the ratio of the maximum bulb diameter at the base to the minimum at the neck—increases to greater than two [8,185]. Bulb formation in temperate areas is photoperiod-reliant, and the leaves of the plant are the photoperiodic stimulus receptor [11,186,187]. Long-day crops are grown in temperate areas and need a minimum of 14 or more hours of light to stimulate bulb beginning, whereas short-day crops grow in additional tropical regions and need a photoperiod of only 10 h or more for bulbing [4]. The substance is more complex, as some cultivars are central and thus require 12 h or more of daylight before they will start producing the bulbs. This photoperiod-reliant bulb beginning is comparable to the photoperiodic regulation of flowering in plants [141,163]. Hence, it is assumed that the genes involved in the photoperiodic regulation of flowering in Arabidopsis are also responsible for the photoperiodic regulation of bulb formation. Both developments are induced by long days; indicator insight is in the leaf blade, response is at the meristem, and both are promoted by far-red light through phytochrome A (PHYA) [27,188]. Rashid et al. [189] conducted an investigation to characterize the advanced and longitudinal expression of supposed photoperiodism-related genes by quantifiable gene expression analysis in different response types of onion under a variety of bulbing and non-bulbing environments so as to further comprehend their possible roles in the photoperiodic parameter of bulbing. Moreover, their research team intended to obtain a better consideration of the molecular regulation of bulbing in response to photoperiod and explicitly examine whether the molecular regulation involved genes controlling flowering by photoperiod in Arabidopsis [189] (Table 1).

Arabidopsis flowering and onion bulbing are both photoperiodically-controlled developmental events [190] that are induced by long days; signal insight lies in the leaf and response is at the top. Sepals, petals, stamens, and anthers are formed as the end produce in Arabidopsis, whereas storage-scale leaves are formed as the end produce in bulbs [191]. Arabidopsis flowering and onion bulbing can be linked via the phytochrome, and both developments are promoted by far-red light through PHYA [192]. Flowering in Arabidopsis has been considered at the molecular and genetic level and is regulated by six major separate pathways viz., photoperiodic, convergent autonomous, sucrose, gibberellin, temperature, and light quality pathways [190,193,194]. For onion, the key ecological stimuli are the photoperiod and temperature [195], but these are mostly grounded on physiological activity more than genetics analyses [196]. Rashid and Thomas [197] emphasized an independent mechanism that generates endogenous rhythms in a 24-h period in the leaf [1] and is controlled by various reaction circles on the photoperiodic pathway, which is intermediated by the circadian clock [198]. Light plays a vital part in the photoperiodic response in Arabidopsis and interrelates with the circadian clock as a fragment of the photoperiodic flowering pathway [199]. In the leaf, light is acknowledged by diverse photoreceptors, including cryptochromes in blue light, phytochromes in red/far-red light, and inputs into the circadian clock [200,201].

## 6. FT Gene Regulates Bulb Formation

The FLOWERING LOCUS T (FT) gene was first recognized in *Arabidopsis thaliana* [202,203] and has been revealed to be the main factor of the floral signal molecule, florigen [175]. FT plays a key role in the photoperiodic pathway for the initiation of flowering in the apical meristem with the help of other floral homeotic genes such as LFY [204]. Moreover, FT is a target of CONSTANS (CO), turns upstream of suppressor of CONSTANS overexpression (SOC1), and can act as a mobile flowering signal to induce flowering by long-distance transportation [163,180]. For bulbing, as with flowering, photoperiod insight emerges in the leaves, while the response is in the meristem [4]. These recommend that a mobile signal with properties parallel to FT might be involved. Additionally, to the regulation of flowering, FT genes have been found to be involved in a range of physiological developments, signifying a more extensive role as a plant hormone [12]. For instance, FT promotes vegetative growth and the inhibition of bud set in poplar in response to warm temperatures and long-day photoperiods [171,205,206] in tomato and maize. In addition, FT genes have been found to function as general growth controllers [207,208]. Other than vegetative growth and flowering, FT is also involved in the short-day initiation of tuberization in potato [170]. Describing genes involved in the photoperiod requirement of bulb formation will help in understanding the basis of the difference between different photoperiod categories, which is important for acclimating novel cultivars for growth and development at diverse latitudes [189] (Table 1) (Figure 1).

The FLOWERING LOCUS T (FT) gene plays a central role in integrating flowering signals in Arabidopsis, because its expression is regulated antagonistically by the photoperiod and vernalization pathways. FT encodes a mobile signaling protein involved in regulating flowering, as well as other aspects of plant development such as bulb formation [12,209]. FT is an important integrator gene and has a significant part in directing the time of evolution to the reproductive phase [202,203,210,211]. The expression of FT genes is inclined by environmental indicators such as the photoperiod, vernalization, hormones, and independent parameters [212,213]. In onion, three FT-like genes are primary groups in directing bulb formation (AcFT1 and AcFT4) and flowering (AcFT2). While flowering is authorized by vernalization and is associated with the up-regulation of AcFT2, a long-day photoperiod is associated with the down-regulation of AcFT4 and up-regulation of AcFT1, which ratifies bulbing [12]. Although warm storage shaped garlic bulb and vegetation stems under long days (12–14 h light) after 80–90 days of growth, the plants from cold storage formed a bulb after only 30 days of growth under short-to-medium days (9–12 h light), and the floral stem was not formed. Furthermore, in nourishment of the early bulbing phenotype after cold storage, gene-expression results established a higher comparative expression of AsFT1 in garlic’s internal bud and in the storage leaf under cold as compared to warm storage. AcFT1 has been suggested to induce bulbing in *Allium cepa*, and the potato FT ortholog StSP6A is involved in the short-day initiation of tuberization [12,163]. AcFT2 is strongly exaggerated by day-length conditions, but the expression outlines confirmed the alterations in bulb enlargement under diverse ecological environments [4]. Manoharan et al. [214] examined that the mRNA levels of AcFT1 genes of EM (Early maturation) and LM (Late maturation) lines of onion were down-regulated when the plants were subjected to both photoperiod environments. Further, AcFT1 and AcFT4 were down-regulated in the EM line under both short-day and long-day environments near bulb maturity, and this was in distinction with the greenhouse environments. Although AcFT4 was up-regulated throughout short-day conditions in the LM line, this might be because of genetic alterations connected to the bulbing period in the two onion lines. Therefore, these fallouts offer additional indications that AcFT4 might be twisted in bulb enlargement and propose that AcFT4 movement might be dependent on internal factors. The functionality of the AcFT genes might be transformed by external factors, for example light intensity and temperature inside the growth room [215]. The LM line reaches a critical long-day length, while AcFT4 and AcFT1 transcription levels are reduced and improved individually once subjected to short-day conditions, therefore encouraging bulb enlargement [12]. Additional research will be required to recognize the instigation and inhibitory action of AcFT4 during short-day and long-day environments in bulbing [215]. Manoharan et al. [214] also anticipated that the enlarged expression of AcFT4 in short-day conditions might also be intricate in the late maturity of the LM line. Bulbing occurs mainly under long-day conditions in onion. An alike outline linked to flowering has been logical in other plants, particularly Arabidopsis, in which FT was up-regulated when plants were visible to long-day photoperiod environments [216]. Tuberization in potato is meticulous by the photoperiod response to short day [217], signifying that the genetic switch is parallel in tuberization. Preceding conclusions evidently display that FT genes are connected to flowering [216]. FT genes are well-maintained in species including rice [218], tomato [209], darnel ryegrass [5], sugar beet [219], and wheat [220].

Genes encrypting FT-like proteins show a main part in monitoring both onion bulb enlargement and flowering. A model of the roles of FT-like genes in the periodic switch of bulb crops growth specified that in juvenile plants and those grown under a non-inductive photoperiod, AcFT4 prevents the up-regulation of AcFT1. Once the plants are mature and the day length holds a risky length, AcFT4 is down-regulated and AcFT1 is up-regulated, leading to the initiation of bulb enlargement. Constant with this prototypical, constitutive expression of AcFT4, AcFT1 up-regulation and bulb enlargement are both prevented. In sugar beet, two FTs with different expression profiles and antagonistic purposes control flowering are involved [219,221]. Rashid et al. [189] conducted an inclusive set of evolving and longitudinal quantifiable mRNA expression trials to examine the expression of onion FLOWERING LOCUS T (AcFT), LEAFY (AcLFY), and GIBBERELLIN-3 OXIDASE (GA3ox1) during the bulbing response. Bulbing proportions were used to measure the response of onion plants under long-day and short-day environments. AcFT1 was expressed in a long-day environment, which influences bulb formation, while AcFT4 was expressed in a short-day environment, which inhibits bulb formation. AcFT5 and AcFT6 were expressed in a long-day environment and might also be involved in bulb formation itself. All AcFT, AcLFY, and GA3ox1 genes presented unique outlines of tissue specific expression in onion, with AcFT genes were found mainly in the locations of insight in the leaf and LFY was found in the basal tissues, which are the place of response. The outcomes are constant with AcFT1 expression being the indicator for long-day influenced bulb instigation and AcFT4 being involved in overwhelming bulbing in short-day environments. According to Rashid and Thomas, [197] onion homologs of CO, FT, GI, and FKF1 genes exhibited diurnal forms of expression in both long-day and short-day onions. The results back their connection in the day-length regulation of bulbing through a mechanism similar to that found in Arabidopsis flowering. Two novel CO-like genes were identified from the RNA-seq library. One of these, AcCOL2, showed an expression pattern very similar to CO from Arabidopsis, which is consistent with a role in day-length regulation. The patterns of mRNA expression presented in this report back the suggestion that AcFT1 encourages bulbing in long-day onions while AcFT4 inhibits bulbing in short-day onions [12]. Furthermore, this study illustrates that these genes are expressed at different times of the day, with AcFT1 expressed in the evening and AcFT4 expressed in the morning. Lyngkhoi et al. [222] stated that in the short-day onion variety Pusa Riddhi, expression of five of the six genes evaluated (AcFT1, AcFT3, AcFT4, AcFT5, and AcFT6) was highest at the bulbing period, signifying their role in bulbing in short-day onions. The expression of AcFT2 was lowest at the bulbing point signifying that the down-regulation of this gene encourages bulbing in the short-day variety. Differing from results observed in the short-day variety, the expression of all the six genes confirmed under investigation was relatively very low at the bulbing stage in Brown Spanish.

Molecular genetic analysis in a wide collection of plants has exposed that sequence variations in mechanisms of the circadian clock [223,224,225,226] and downstream mechanisms [226], including FT genes [227,228], stimulate the difference in day length that is mandatory to influence flowering. Therefore, it is likely that the variation of bulb at the suitable time in diverse latitudes comprises similar mechanisms. Leek is a significant Allium crop that does not contain bulbs and consequently privations a photoperiodic obligation [229,230,231,232,233,234,235]. These landscapes let leeks be implanted during the whole season and fully grow over a wide range of latitudes. Attractively, the 35S:AcFT4 plants that do not bulb have a similar presence to leeks and sustain budding vegetatively into the winter, despite being deprived of their vegetation dying off. This recommends that the large phenotypic alterations between onion and leek might be due to an uncertain genetic variation [236,237,238,239,240,241]. Although the occurrence of enlarged bulbs is a distinct feature of onions, most members of the Allium genus produce some kind of storage organ. For instance, garlic cloves (the storage organ) are formed from inflamed bladeless internal sheaths, but distinct to onion, no storing happens in foliage leaf bases, while chive storage is in foliage leaf bases; nonetheless, the bulbs are indefinite. It will be exciting to determine the role that FT-like genes play in other Alliums and whether genetic discrepancy within FT genes, or their goals, clarifies their phenotypic difference. In short, FT genes control both bulb enlargement and floral initiation. It too enhances the growing body of indication that FT genes not only solitary control flowering, but they also play a broader part in monitoring developing selections [242,243,244,245,246,247].

## 7. Conclusions

Substantial progress has been made in understanding the mechanisms regulating the initiation of bulb enlargement in plants. Several genes have been identified to control bulb crops, and alterations of their expression levels confirms the regulation of bulbing. Critical detections have been the outcome that clock mechanisms and a CO-FT component connected to that testified in crops such as Arabidopsis are concerned in the day-length switch of bulb enlargement, while as high gibberellins persuade the enlargement of these tissues in types where this development has been evolutionarily stifled. Bulbs can be tempted in axillary buds of stem node models, and it is being established that phytohormones play an imperative role in axillary meristem instigation and obligation for bulb growth and enlargement. Florigen FT proteins control the axillary shoot branching, and it will be vital to evaluate if extra FT family affiliates are similarly concerned in the bulbing modification. It will be interesting to examine if bulb initiation by high-temperature frameworks demonstrate leading fundamentals with the photoperiod regimes. More importantly, bulb growth and development in geographical regions with low temperatures that pacified the cultivation of these bulb crops might be interesting. Furthermore, the main emphasis of future research should be on the identification of genes and gene products controlling the bulbing parameters under different photoperiodic environments. Identification of both host as well as photoperiodic specific protein factors regulating the synergetic association and the key cellular and metabolic pathways under different photoperiodic conditions can be hot areas for future research. Understanding the photoperiodic-induced modulations in the bulbing mechanisms and the cross-talk mechanism triggered to regulate the bulbing performance can help improve crop productivity. Taken together, the photoperiod must be explored at all levels to further investigate their role in nature as an environmental cue for managing and improving horticultural production.

## Figures and Tables

**Table 1:** Bulbing response to the photoperiod and its mechanism.

Highlights
Bulb enlargement and its subsequent development were influenced by photoperiod and bulbing was encouraged by long days [2,3].
Bulbing is regulated by internal signals, which can be stimulated or inhibited by the environmental conditions. It has been widely reported that phytohormones regulate the plant growth and are considered to play an important role in the formation of bulbs [5,7].
Long photoperiods are known to improve the levels of endogenous gibberellins, with consequent flower bud differentiation. Many studies have shown that gibberellic acid (GA) could partially or fully replace vernalization for some plants. Endogenous GA levels of long day or biennial plants during the process of floral induction increased. However, GA is considered to be an inhibitor of bulb formation. Exogenous GA inhibited the increase of the scape and bulb yield. It was likely that GA did not act directly on the inhibition of bulbing; instead, it enhanced the activity of a “bulbing inhibition substance” [5,7].
Abscisic acid (ABA) generally plays an important role in plant defense against biotic or abiotic stresses. It was assumed that ABA acts similarly to GA in the early stage of plant bolting. Endogenous ABA levels of Welsh onion (*Allium fistulosum* L.) increased significantly during flower bud differentiation and decreased dramatically after the completion of flower bud differentiation [5,7]
Indoleacetic acid (IAA) showed the opposite effect, decreasing with increases in the flower bud differentiation rate but increasing significantly during the bolting process of Welsh onion. It is reasonable to assume that IAA inhibits flower bud differentiation but improves plant bolting [5,7].
Zeatin riboside (ZR) also showed an enhancing effect on plant bolting. Cytokinin (CTK) was a bulbing initiator but had no visible effect on bulb enlargement, while IAA and ethylene improved bulb formation. However, few studies have investigated the role of abscisic acid (ABA) on garlic bolting or bulbing [5,7].
Jasmonic acid (JA) and related compounds are widely distributed among higher plants and play important roles in the regulation of plant development. It was found that jasmonates were potent inducers of vegetative storage protein gene expression and proteinase inhibitors of defense proteins. It is generally believed that the bulbing process is regulated by the balance between the “bulbing hormones” and GA. By considering that bulbing was involved in the disruption of microtubules, jasmonic acid (JA) and methyl jasmonate (MeJA) were candidate bulbing hormones because of their microtubule-disrupting activities and wide distribution in higher plants [5,7].
Salicylic acid (SA) played an important role in garlic bulb formation and MeJA likely enhanced the endogenous SA content of garlic plant, thus improving bulbing [5,7].
Cultivars grown at diverse latitudes required a least day length for bulbing, and cultivars are classified on this into short-day (SD), intermediate, and long-day (LD) categories. The short-day cultivars procedure bulbs at low latitudes whenever the day length is close to 12 h, whereas intermediate ones grow bulbs at mid latitudes whenever the day length lies between 12 and 16 h, and long-day cultivars initiate bulbing at high latitudes whenever the day length is close to or above 16 h [6,7,8,9,10,11].
Numerous key genes are intricate in circadian regulation, where the clock derives the rhythmic expression of key genes FLAVIN-BINDING, KELCH REPEAT, F-BOX (FKF1), GIGANTEA (GI), and CONSTANS (CO). FKF1 and GI promote CO expression and this CO positively controls FLOWERING LOCUS T (FT). Then, the FT protein is translocated to the apical meristem through the phloem and forms a FT/FD (FLOWERING LOCUS D) complex. This compound triggers the APETALA 1 (AP1) and suppressor of overexpression of CONSTANS 1 (SOC1) genes, which triggers LEAFY (LFY) gene expression and causes flowering at the floral apical meristem in Arabidopsis. The expression of GI, FKF1, and ZTL homologs under short-day and long-day environments was observed using quantitative reverse transcription-PCR (qRT-PCR), where the results presented that key genes—namely GI, CO, and FT—controlling photoperiodic flowering in Arabidopsis are conserved in Alliums, and a role for these genes in the photoperiodic control of bulb instigation is anticipated [12,13].
The FLOWERING LOCUS T gene (FT), which was first documented in Arabidopsis thaliana, has been discovered to be the main feature of the floral signal molecule florigen. FT plays a key role in the photoperiodic pathway for the initiation of flowering in the apical meristem with the help of other floral homeotic genes such as LFY. Moreover, FT is a target of CONSTANS (CO) and turns upstream of suppressor of CONSTANS overexpression (SOC1) and can act as a mobile flowering signal to encourage flowering by long-distance transport. For bulbing, as with flowering, photoperiod insight develops in the leaves, while the response is in the meristem. These indorse that a mobile signal with properties similar to FT might be involved [12,13].
